# Dynamic Reconstruction‐Engineered Heterointerfaces for Acidic Hydrogen Evolution at Ampere‐Level Current Density

**DOI:** 10.1002/advs.76032

**Published:** 2026-06-12

**Authors:** Kaixi Wang, Yunshan Zheng, Chengzong Yuan, Kwun Nam Hui

**Affiliations:** ^1^ Joint Key Laboratory of the Ministry of Education Institute of Applied Physics and Materials Engineering University of Macau Avenida da Universidade Taipa China; ^2^ Zhengzhou Advanced Research Institute Harbin Institute of Technology Zhengzhou China; ^3^ Jiangxi Province Key Laboratory of Cleaner Production of Rare Earths Ganjiang Innovation Academy Chinese Academy of Sciences Ganzhou China

**Keywords:** acidic hydrogen evolution, ampere‐level current density, copper phosphide nanowires, dissolution‐redeposition equilibrium, dynamic reconstruction

## Abstract

The industrial application of proton exchange membrane electrolysis for hydrogen production urgently requires acidic hydrogen evolution reaction (HER) catalysts that combine high activity with long lifespan at ampere‐level current densities. This work employs electrochemically driven dynamic reconstruction to in situ transform self‐supporting Cu_3_P nanowires into a core–shell structure with a loose surface layer consisting of PtCu/Cu_3_P heterostructures. The reconstructed catalyst exhibits benchmark HER performance in acid, requiring low overpotentials of only 37.8 and 207.8 mV at 10 and 1000 mA cm^−2^, respectively, while maintaining stable operation for over 240 h at 1000 mA cm^−2^. Mechanistic studies reveal that the heterointerface synergistically regulates hydrogen adsorption free energy and facilitates proton transport, while the loosely porous morphology formed by reconstruction ensures efficient mass transfer at high currents. The catalyst's exceptional durability stems from the dynamic dissolution‐redeposition equilibrium of surface metal species during operation. This work reveals a novel reconstruction pathway in acid involving foreign metal participation, enabling industrial‐scale acidic HER catalysis.

## Graphical abstract

A dynamic reconstruction strategy was utilized to construct PtCu/Cu_3_P heterostructures on the surface of self‐supporting Cu_3_P nanowires. The loose porosity in the restructured layer, together with a dynamic “dissolution‐redeposition” equilibrium during electrocatalysis, enables the electrode to maintain a low, stable overpotential for 240 h at ampere‐level current density in acidic conditions, outperforming commercial Pt/C.

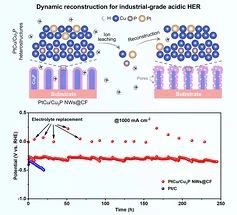

## 1 Introduction

Hydrogen production through water electrolysis powered by renewable electricity is a key pathway for achieving green hydrogen production [1, 2, 3, 4, 5]. Among various technologies, proton exchange membrane (PEM) electrolyzers are considered one of the most promising pathways due to their high efficiency (∼70%), sub‐second dynamic response, high system integration, and capability for ampere‐level current density operation [6, 7]. However, its commercialization has long been constrained by the high dependence on platinum‐group metal catalysts in acidic environments. Although Pt/C is still the performance benchmark for acidic hydrogen evolution reaction (HER), extensive research has focused on developing low‐platinum or platinum‐free alternatives. Yet the vast majority of these materials demonstrate stability only at low current densities (<100 mA cm^−2^) [8], making them unsuitable for the demanding conditions of industrial electrolysis. In recent years, self‐supporting catalysts grown directly onto conductive substrates have demonstrated significant potential to address this challenge [9]. This integrated design not only avoids the problems of ion transport resistance, active site masking, and poor interface contact caused by traditional polymer binders [10], but also effectively accelerates bubble desorption and suppresses gas film formation by finely regulating surface morphology and hydrophilicity, thereby alleviating common mass transfer bottlenecks and Ohmic losses under high current [11, 12]. Therefore, self‐supporting electrodes demonstrate unique potential for achieving low overpotential and long‐term stable operation under harsh ampere‐level HER conditions.

The dynamic restructuring of catalysts under electrochemical action is recognized as a core factor determining their catalytic performance [13, 14]. Under actual operating conditions, the chemical composition, oxidation state, crystalline phase, and microstructure of materials often undergo irreversible or reversible transformations in response to harsh reaction environments. It is this adaptive process that fundamentally shapes their intrinsic activity and stability [15]. The catalysts for oxygen evolution reaction (OER) are by far the most extensively documented in the literature as undergoing structural reconstruction, which stems from the inherent thermodynamic instability of most transition metal‐based materials under highly oxidizing OER potentials, and they tend to reach a relatively stable state through dynamic structural evolution [16]. Beyond OER, reconstruction phenomena have also been documented in other key electrocatalytic processes, including the hydrogen evolution reaction (HER) [17], CO_2_ reduction [18], and nitrate reduction [19].

Specifically for the HER, a range of materials have been discovered to undergo self‐reconstruction processes under reductive potentials, including sulfides [20], oxides [21], fluorides [22, 23], phosphides [24, 25, 26], borides [27], and oxalates [28, 29]. The reconstructed species (metal oxides or hydroxides, etc.) can act synergistically with the initial components to modulate the adsorption behavior of key reaction intermediates, thereby optimizing the intrinsic catalytic activity [30]. However, almost all reported research has focused on the reconstruction process in alkaline or neutral working conditions. In contrast, the landscape of catalyst reconstruction under the aggressive conditions of acidic HER remains remarkably underexplored and poorly understood. The different chemical environment (e.g., high H^+^ concentration) likely drives distinct reconstruction pathways and yields different active species compared to the alkaline system, which provides an effective strategy for the design of acidic HER catalysts. Bearing these points in mind, it is greatly attractive to utilize the structural reconstruction of self‐supported electrocatalysts to develop highly active and durable low‐Pt cathodes capable of sustaining ampere‐level current densities.

In this work, we focus on the structural evolution of self‐supporting Cu_3_P nanowires under acidic hydrogen evolution conditions. Interestingly, unlike conventional reconstruction processes that are governed by external potential and electrolyte chemistry, the dissolved Pt species from the counter electrode in this system actively participate, leading to the formation of a loosely structured heterointerface layer, where PtCu alloy nanoparticles are anchored to the Cu_3_P framework. In situ characterization combined with theoretical calculations indicates that the PtCu/Cu_3_P heterointerface effectively optimizes hydrogen binding energy and facilitates proton transfer, while the loose and porous reconstructed surface enhances mass transport. Leveraging these advantages, the restructured catalyst achieves highly efficient hydrogen evolution performance in 0.5 m H_2_SO_4_, requiring overpotentials of only 37.8 mV (at 10 mA cm^−2^) and 207.8 mV (at 1000 mA cm^−2^). More importantly, it maintains stable operation at 1000 mA cm^−2^ for more than 240 h. This exceptional ampere‐level durability is attributed to a dynamic dissolution‐redeposition equilibrium occurring on the surface of the reconstructed catalyst.

## 2 Results and Discussion

**FIGURE 1:**
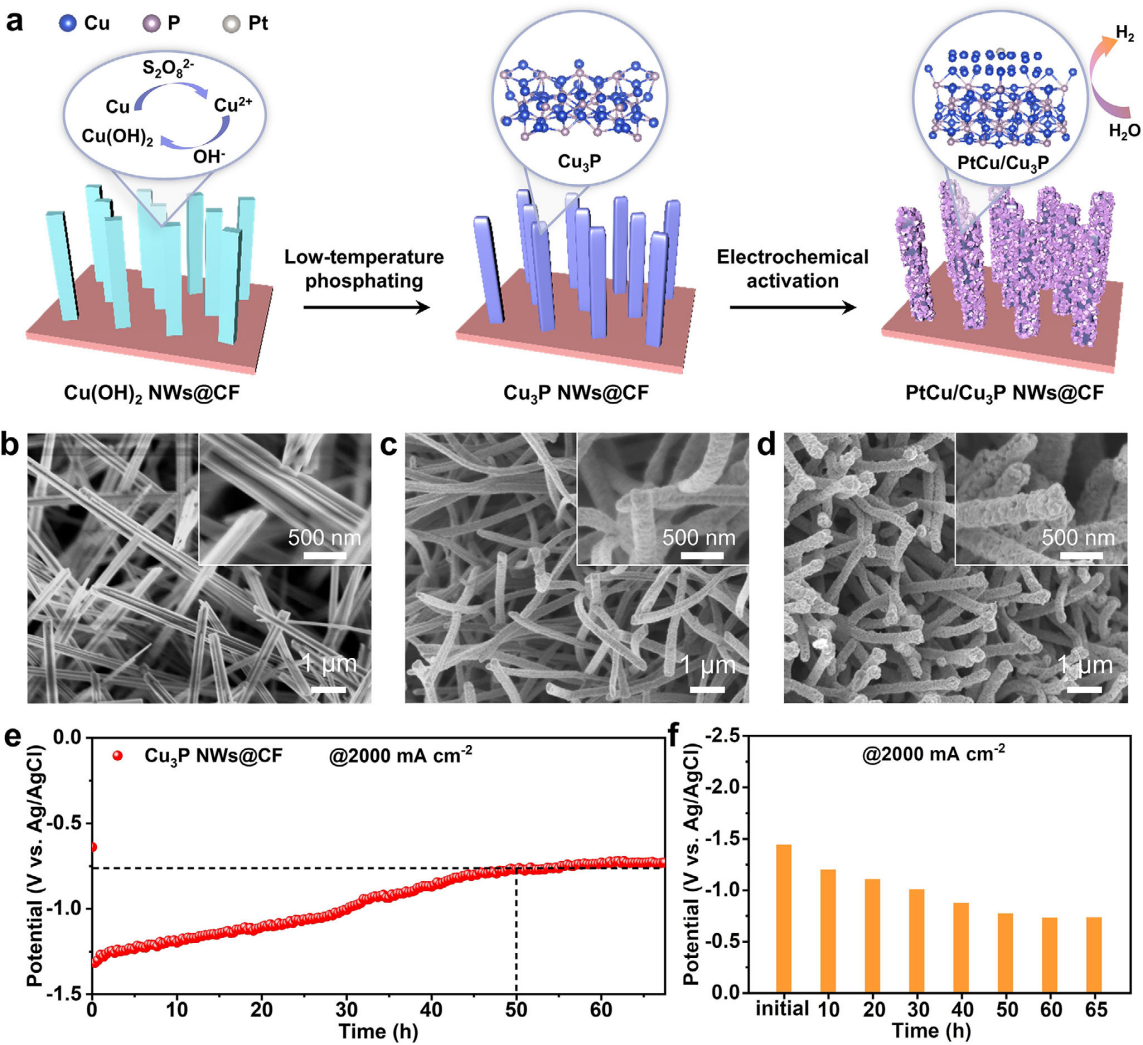
(a) Schematic representation of the synthetic protocol of PtCu/Cu_3_P NWs@CF. SEM images of (b) Cu(OH)_2_ NWs@CF, (c) Cu_3_P NWs@CF, and (d) PtCu/Cu_3_P NWs@CF. The insets are the SEM images at higher magnification. (e) Electrochemical activation of Cu_3_P NWs@CF with the chronopotentiometry at 2000 mA cm^−2^. (c) The potential change with the activation time for Cu_3_P NWs@CF at 2000 mA cm^−2^.

The fabrication strategy for PtCu/Cu_3_P NWs@CF is schematically illustrated in Figure 1a. Initially, a self‐supported Cu(OH)_2_ nanowire array was grown on the smooth surface of Cu foam (Figure S1) via a wet chemical oxidation reaction (Figure 1b and Figure S2). The related reaction equation is: Cu + 4 NaOH + (NH_4_)_2_S_2_O_8_ → Cu(OH)_2_ + 2 Na_2_SO_4_ + 2 NH_3_ + 2 H_2_O [31]. Transmission electron microscopy (TEM) and high‐resolution TEM (HRTEM) characterization clearly decipher that the nanowires are rich in mesopores, exhibiting a distinct lattice fringe spacing of 0.24 nm consistent with the (041) crystal planes of Cu(OH)_2_ (Figure S3). Subsequently, Cu(OH)_2_ NWs@CF was directly transformed into Cu_3_P NWs@CF through a low‐temperature phosphating process using a three‐zone tube furnace (Figure S4). Specifically, the phosphorus source (NaH_2_PO_2_) was placed in zone 3, which was heated to 290°C, while zones 1 and 2 were set to room temperature. During the phosphating process, Cu(OH)_2_ NWs@CF (located in zone 1) experienced an indirect temperature increase to approximately 153 °C due to the flow of hot N_2_ and PH_3_ originating from zone 3, thereby enabling a mild phosphidation process.

Post‐phosphidation scanning electron microscopy (SEM) images reveal that the nanowire architecture of Cu_3_P NWs is well preserved, with diameters comparable to those of the original Cu(OH)_2_ NWs, but their surface becomes noticeably rougher (Figures 1c and S5). The morphological evolution was further examined by TEM, which shows the emergence of numerous surface convex features (Figure S6a–c), consistent with the SEM observations. The lattice spacing was measured as 0.25 nm, which aligns closely with the (111) lattice planes of crystalline Cu_3_P (Figure S6d). The successful formation of Cu_3_P NWs@CF was unequivocally demonstrated by x‐ray diffraction (XRD) and x‐ray photoelectron spectroscopy (XPS) measurements (Figures S5d and S7). It should be noted that the formation of Cu_3_P NWs is highly sensitive to both the amount of NaH_2_PO_2_ and the phosphating temperature. An insufficient amount of NaH_2_PO_2_ can lead to incomplete transformation of Cu(OH)_2_ (Figures S8a–c and S9), while an excessive amount of NaH_2_PO_2_ results in larger diameters of the obtained nanowires (Figure S8d–f). Additionally, diminishing the distance between the precursor and the phosphorus source, which implies an increase in phosphorization temperature, also results in larger diameters of Cu_3_P NWs (Figure S8g–i). When the precursor‐phosphorus source distance is minimized, the nanowires will aggregate together to form larger particles (Figure S8j–l). All these preparation conditions will result in reduced HER activity of the obtained Cu_3_P NWs@CF (Figure S10).

The obtained Cu_3_P NWs@CF was subjected to electrochemical activation in 0.5 m H_2_SO_4_ using a three‐electrode configuration, with the Cu_3_P NWs@CF and a platinum foil serving as the working and counter electrodes, respectively (Figures S11 and S12). Chronopotentiometric measurement was conducted by applying a cathodic current density maintained at 2000 mA cm^−2^. During the activation, the potential vs. Ag/AgCl gradually decreased from −1.44 to −0.76 V and stabilized after 50 h (Figure 1e,f), indicating a pronounced enhancement in the electrocatalytic activity of the catalyst. Therefore, activation for 50 h was selected as the optimal time, and the in situ generated product is denoted as PtCu/Cu_3_P NWs@CF. The SEM images show that PtCu/Cu_3_P NWs@CF still maintains an intact self‐supporting nanowire morphology, but the surface becomes obviously rougher (Figures 1d and S13). In addition, many stacking holes were created on the surface of the nanowires. This unique hierarchical nanowire array configuration of PtCu/Cu_3_P NWs@CF can provide a more efficient pathway for mass and charge transport during HER, contributing to an optimal performance of PtCu/Cu_3_P NWs@CF in HER [32, 33]. These results suggest that during the electrochemical activation, the nanowires underwent reconstruction and some Pt species may have been deposited onto the nanowires [34]. To verify this hypothesis, we changed the Pt counter electrode to a graphite rod while keeping all other conditions constant, and the obtained samples are denoted as Cu_3_P NWs@CF‐Gr. It can be seen that the potential of the Cu_3_P NWs@CF‐Gr electrode remained almost unchanged after 50 h of activation at −2000 mA cm^−2^ (Figure S14). In addition, Cu_3_P NWs@CF also experienced a more severe reconstruction in the process (Figure S15), which may be caused by the fact that Cu_3_P NWs@CF has been suffering from a greater polarization potential. The above comparative experiments suggest that the improved performance of PtCu/Cu_3_P NWs@CF is attributed to the modification of Pt species. Then, XRD was used to probe the crystal structure of PtCu/Cu_3_P NWs@CF. The diffraction peaks associated with Cu_3_P become weaker due to the partial loss of P species during the acidic HER process (Figure S16) [35]. The peaks from Cu_2_O are also observed, which should be formed by the exposure of active Cu to air. It should be noted that Cu_2_O is not stable in acid and will undergo a disproportionation reaction to generate Cu and Cu^2+^ (Cu_2_O + H_2_SO_4_→CuSO_4_ + Cu + H_2_O). Besides, there is a small diffraction peak at 40.0° positioned between the standard peaks of Pt and Cu, suggesting the formation of PtCu alloy in the electrodeposition and reconstruction processes [36].

**FIGURE 2:**
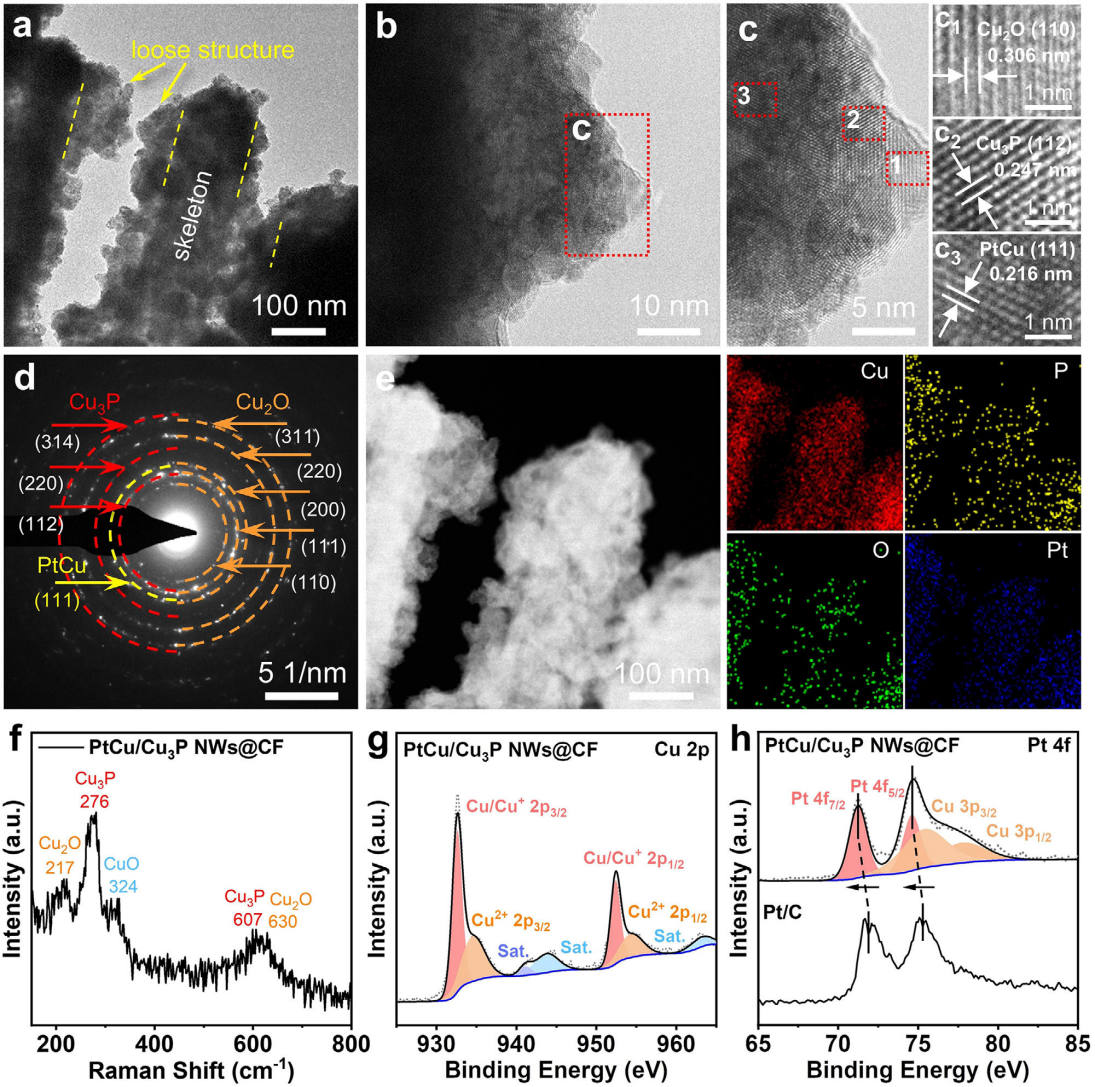
(a, b) TEM and (c) HRTEM images of PtCu/Cu_3_P NWs@CF. (c_1_–c_3_) Magnified HRTEM images from the red‐dashed regions indicated in (c). (d) SAED pattern, (e) HAADF‐STEM image with the corresponding EDS elemental mapping images, (f) Raman spectrum, and (g) Cu 2p XPS spectrum of PtCu/Cu_3_P NWs@CF. (h) Pt 4f XPS spectra of PtCu/Cu_3_P NWs@CF and commercial Pt/C.

To elucidate the morphology and structural characteristics of the PtCu/Cu_3_P NWs@CF sample, TEM, and HRTEM analyses were conducted. The material exhibits a nanowire framework with a loosely textured surface (Figure 2a), composed of numerous aggregated nanoparticles (Figure 2b and Figure S17). The HRTEM image (Figure 2c) clearly resolves lattice spacings of 0.306 and 0.247 nm, which correspond to the (110) plane of Cu_2_O (Figure 2c1) and the (112) plane of Cu_3_P (Figure 2c2), respectively. Moreover, an additional lattice fringe of 0.216 nm (Figure 2c3) lies between the characteristic spacings of the (111) planes of metallic Cu (0.208 nm) and Pt (0.227 nm), confirming the formation of a PtCu alloy on the reconstructed surface. This interpretation is corroborated by the selected area electron diffraction (SAED) pattern (Figure 2d), which displays diffraction rings corresponding to Cu_2_O, Cu_3_P, and PtCu alloy, in agreement with XRD and HRTEM findings. Furthermore, high‐angle annular dark‐field scanning TEM (HAADF‐STEM) image and corresponding energy‐dispersive x‐ray spectroscopy (EDS) mappings confirm a uniform spatial distribution of Cu, P, O, and Pt across the nanostructure (Figure 2e). Quantitative analysis via inductively coupled plasma‐atomic emission spectrometry (ICP‐AES) determined the Pt mass loading on the PtCu/Cu_3_P NWs@CF electrode as 266 µg cm^−2^.

The surface composition of the catalyst was determined by Raman spectroscopy and XPS analysis. Figure 2f displays the Raman spectrum of PtCu/Cu_3_P NWs@CF. The characteristic peaks at 276 and 607 cm^−1^ are indicative of Cu_3_P [37], while signals at 217 and 630 cm^−1^ correspond to Cu_2_O phases [38]. An additional peak at 324 cm^−1^ is assigned to CuO surface species [39]. The full XPS spectrum demonstrates the presence of Cu, P, O, and Pt in PtCu/Cu_3_P NWs@CF (Figure S18a), in agreement with the EDS results. The high‐resolution Cu 2p spectrum (Figure 2g) reveals two primary components, with the peaks appearing at 932.6 and 952.4 eV, which are associated with Cu/Cu^+^, whereas the alongside peaks at 934.7 and 954.4 eV are typically ascribed to Cu^2+^ [32, 40]. Compared to the Cu 2p spectrum of Cu_3_P NWs@CF in Figure S7b, the Cu^2+^ ratio in PtCu/Cu_3_P NWs@CF is markedly reduced with a corresponding increase in the Cu/Cu^+^ ratio. This is likely due to the dissolution of the oxidized copper on the Cu_3_P surface as well as the formation of some Cu_2_O during preparation. Deconvolution of the P 2p spectrum (Figure S18b) yields two contributions centered at 129.0 and 133.7 eV, signifying the presence of metal phosphides and oxidized phosphorus species on the surface of PtCu/Cu_3_P NWs@CF [40, 41]. In the O 1s region (Figure S18c), the signal at 531.7 eV can be attributed to lattice oxygen in Cu_2_O [42]. Notably, the electronic structure of Pt decisively influences the HER activity. Figure 2h shows that the Pt 4f spectrum of PtCu/Cu_3_P NWs@CF exhibits a double peak at 71.2 and 74.6 eV, corresponding to the 4f_7/2_ and 4f_5/2_ orbitals of metallic Pt [43]. Compared to commercial Pt/C, both peaks display a significant shift toward lower binding energies, indicating substantial electron density enrichment of Pt within the reconstructed structure. Concurrently detected Cu 3p peaks were fitted at 75.5 eV (Cu 3p_3/2_) and 77.9 eV (Cu 3p_1/2_), in line with metallic Cu [34]. Collectively, these findings provide further evidence for the successful construction of a PtCu/Cu_3_P heterointerface with charge redistribution in PtCu/Cu_3_P NWs@CF, which is favorable for regulating the adsorption of hydrogen intermediates.

To further elucidate the dynamic reconstruction process during electrochemical activation, interruption experiments were performed by collecting intermediate samples after 5, 20, and 35 h of activation, denoted as Cu_3_P NWs@CF‐5 h, Cu_3_P NWs@CF‐20 h, and Cu_3_P NWs@CF‐35 h, respectively. XRD results show that these intermediate samples exhibit phase features similar to those of the fully activated PtCu/Cu_3_P NWs@CF, while the diffraction peak at 40.0° is assigned to the PtCu alloy and becomes progressively stronger with increasing activation time (Figure S19), indicating the gradual formation of more PtCu species. SEM observations reveal that the nanowire morphology is well preserved throughout the activation process, whereas the surface stacked pores become increasingly abundant, suggesting an intensified surface reconstruction (Figure S20). In parallel, SEM‐EDS analysis shows a gradual increase in the Pt/Cu atomic ratio with activation time (Table S1), confirming the continuous deposition of Pt species onto the cathode. TEM analysis further shows that the thickness of the loose reconstructed surface layer increases gradually as activation proceeds, and HAADF‐STEM elemental mapping verifies the uniform distribution of Cu, P, O, and Pt within the reconstructed region (Figure S21). In good agreement with these structural evolutions, the HER activity is continuously improved with increasing activation time (Figure S22a). In particular, the overpotentials at 10 mA cm^−2^ decrease from 140.5 to 90.5 and then to 59.4 mV for Cu_3_P NWs@CF‐5 h, Cu_3_P NWs@CF‐20 h, and Cu_3_P NWs@CF‐35 h, respectively (Figure S22b). Meanwhile, the charge‐transfer resistance decreases markedly with prolonged activation (Figure S22c), indicating accelerated electron‐transfer kinetics. These results collectively demonstrate that Cu_3_P NWs undergo continuous surface reconstruction during electrochemical activation, leading to the gradual formation of a more porous and loose surface structure together with increasing Pt species deposition, which jointly contribute to the enhancement of HER activity.

**FIGURE 3:**
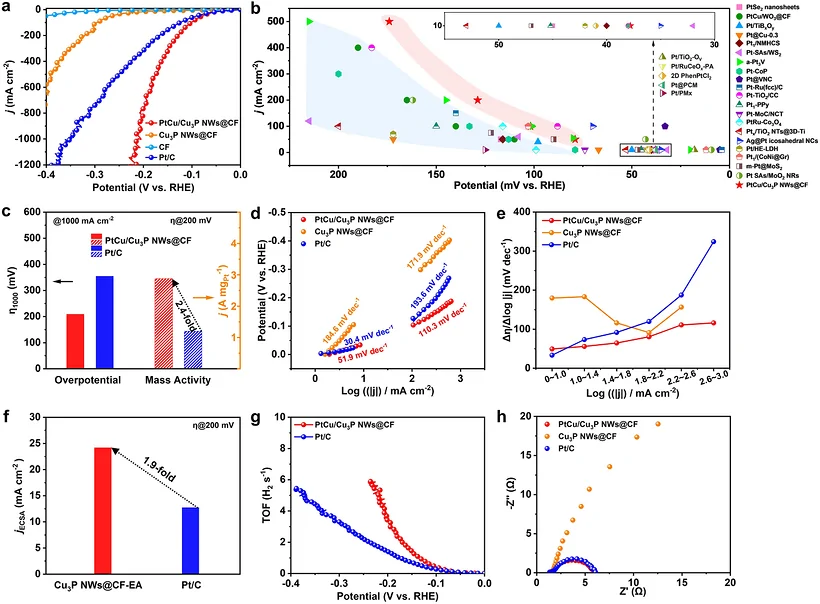
(a) HER polarization curves of PtCu/Cu_3_P NWs@CF, Cu_3_P NWs@CF, CF, and Pt/C in 0.5 m H_2_SO_4_. (b) Comparison of HER activity among PtCu/Cu_3_P NWs@CF and the reported Pt‐based catalysts. (c) Comparison of overpotentials and mass activities between PtCu/Cu_3_P NWs@CF and Pt/C. (d) Tafel plots under low and high current density regimes. (e) Δη/Δlog|j| values as a function of current density. (f) *j*_ECSA_ values at the overpotential of η = 0.2 V. (g) TOF for PtCu/Cu_3_P NWs@CF and Pt/C. (h) Nyquist plots for all four samples.

The electrocatalytic HER activity of PtCu/Cu_3_P NWs@CF catalyst was initially assessed in 0.5 m H_2_SO_4_ using a standard three‐electrode configuration, with Cu_3_P NWs@CF, bare CF, and commercial Pt/C as reference benchmarks. As displayed in the representative polarization curves in Figure 3a, PtCu/Cu_3_P NWs@CF requires an extremely low overpotential of 37.8 mV at 10 mA cm^−2^, substantially outperforming both Cu_3_P NWs@CF (155.8 mV) and CF (306.9 mV) and closely matching the benchmark Pt/C catalyst (35.7 mV). Remarkably, the HER activity of the as‐designed PtCu/Cu_3_P NWs@CF rapidly exceeds that of Pt/C as the current density beyond 15 mA cm^−2^. In addition, it achieves current densities of 500 and 1000 mA cm^−2^ at overpotentials of only 174.0 and 207.8 mV, respectively, significantly 78.6 and 146.5 mV lower than Pt/C. Notably, the increase in overpotential from 500 to 1000 mA cm^−2^ is merely 33.8 mV, underscoring the low kinetic barrier of the electrode at large current density. Figure 3b shows the comparison of HER activity among PtCu/Cu_3_P NWs@CF and the reported Pt‐based electrocatalysts. It can be seen that PtCu/Cu_3_P NWs@CF exhibits a remarkable superiority over most of the documented catalysts at various current densities. To further evaluate Pt utilization efficiency, we normalized the current density according to the Pt loading and calculated the corresponding mass activity. Figure S23 shows that the PtCu/Cu_3_P NWs@CF exhibit higher mass activity than Pt/C. Specifically, at an overpotential of 200 mV, its mass activity reaches 2.88 A mg_Pt_^−1^, which is 2.4 times that of Pt/C (Figure 3c).

Tafel slope can effectively reflect the kinetics of HER on the electrocatalyst surface. The Tafel plots in Figure 3d were calculated based on the polarization curves in Figure 3a. At low current densities (1–10 mA cm^−2^), Pt/C achieves a Tafel slope of 30.4 mV dec^−1^, which is 21.5 mV dec^−1^ lower than PtCu/Cu_3_P NWs@CF (51.9 mV dec^−1^), indicating faster HER kinetics for Pt/C under mild operating conditions. In stark contrast, within the high current density window of 100–1000 mA cm^−2^, PtCu/Cu_3_P NWs@CF demonstrates a markedly lower Tafel slope of 110.3 mV dec^−1^, significantly lower than the 193.6 mV dec^−1^ of Pt/C (a decrease of 83.3 mV dec^−1^), disclosing its superior HER kinetics under high current densities. Subsequently, the HER activities of different electrocatalysts across a wide range of current densities were evaluated by analyzing the slope of the polarization curve Δη/Δlog|j| (R_η/j_) (Figure 3e) [44, 45]. As the current density increases from 1 to 1000 mA cm^−2^, the R_η/j_ of Pt/C electrode rises sharply from 33.5 to 324.3 mV dec^−1^. In contrast, the R_η/j_ of PtCu/Cu_3_P NWs@CF undergoes only a mild change from 49.5 to 116.3 mV dec^−1^, indicating that it exhibits better mass transfer properties under high current densities. Notably, even the Cu_3_P NWs@CF shows a lower R_η/j_ than that of Pt/C at large current densities. This is attributed to its self‐supporting nanowire arrays and the three‐dimensional macroporous structure of CF that promotes charge and mass transfer. Taken together, these results confirm that the unique structure of the PtCu/Cu_3_P NWs@CF electrode enables it to work well at both low and high current densities.

To exclude geometric influences, we measured the electrochemically active area (ECSA) using double‐layer capacitance (C_dl_) and normalized the catalyst activity accordingly [16]. The C_dl_ value was calculated by collecting cyclic voltammetry (CV) curves at different scan rates within the non‐Faradic region at regular intervals of increasing scan rates (Figure S24). As illustrated in Figure S25, both PtCu/Cu_3_P NWs@CF and Cu_3_P NWs@CF have similar C_dl_ values, which are higher than Pt/C. Correspondingly, the ECSA of PtCu/Cu_3_P NWs@CF, Cu_3_P NWs@CF, and Pt/C was calculated to be 31.7, 33.9, and 24.9 cm^2^, respectively (Figure S26a). Notably, PtCu/Cu_3_P NWs@CF shows the highest *j*_ECSA_ compared with the reference samples (Figure S26b). For instance, at an overpotential of 200 mV, its specific activity is up to about 1.9 times as compared to Pt/C (Figure 3f), highlighting its superior intrinsic catalytic activity. Furthermore, the Pt‐based turnover frequency (TOF) is calculated (Figure 3g). It is evident that the PtCu/Cu_3_P NWs@CF owns the highest TOF across the potential range. Specifically, the TOF of PtCu/Cu_3_P NWs@CF reaches 3.4 s^−1^ at an overpotential of 200 mV, while only 1.4 s^−1^ is observed for Pt/C (Figure S27), indicating the higher catalytic efficiency of Pt sites in PtCu/Cu_3_P NWs@CF. Moreover, the enhanced interfacial charge transfer kinetics of PtCu/Cu_3_P NWs@CF are also corroborated by Nyquist plots in Figure 3h. It is noted that PtCu/Cu_3_P NWs@CF displays the smallest charge transfer resistance (R_ct_), implying accelerated electron transport and improved HER kinetics at the electrode–electrolyte interface.

**FIGURE 4:**
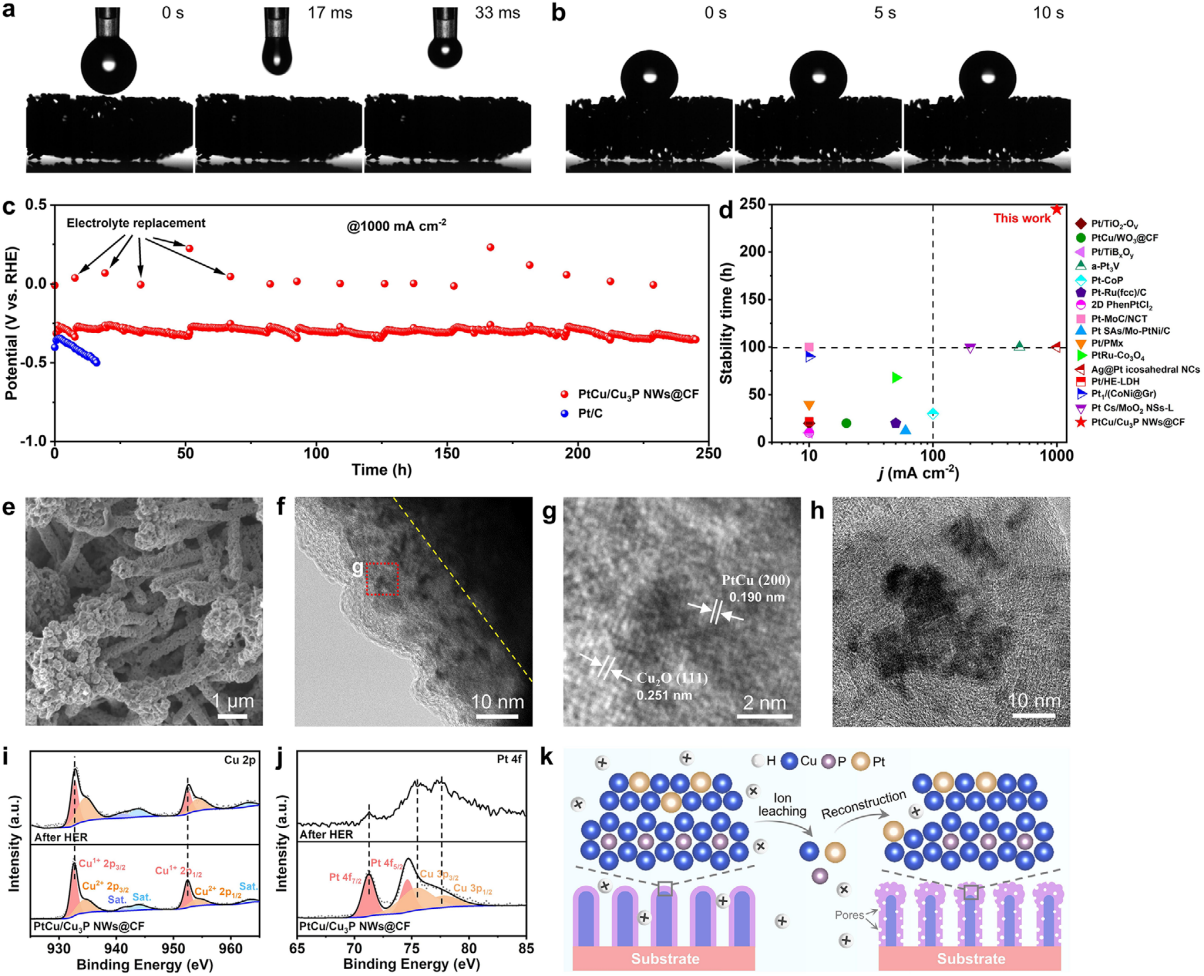
Dynamic contact angle measurement using 0.5 m H_2_SO_4_ for (a) PtCu/Cu_3_P NWs@CF and (b) Pt/C@CF. (c) Chronopotentiometry response of PtCu/Cu_3_P NWs@CF and Pt/C at 1000 mA cm^−2^. (d) Performance comparison between PtCu/Cu_3_P NWs@CF and documented catalysts for acidic HER. (e) SEM, (f) TEM, and (g) HRTEM images of PtCu/Cu_3_P NWs@CF after the stability test. (h) TEM image of Pt/C after stability test. (i, j) XPS spectra of Cu 2p and Pt 4f of PtCu/Cu_3_P NWs@CF before and after the stability test. (k) Schematic depicting the electrochemical dissolution of ions in PtCu/Cu_3_P NWs@CF and the reconstruction process.

During water electrolysis at high current densities, mass transfer processes often become the primary kinetic limiting factor. The surface wettability of the electrode can effectively regulate the reaction environment at the solid–liquid interface, making it a key parameter for evaluating mass transfer capabilities. Therefore, dynamic contact angle measurements were performed using 0.5 m H_2_SO_4_ solution. Figure 4a shows that the droplet rapidly spreads upon contact with the PtCu/Cu_3_P NWs@CF surface, indicating its superhydrophilicity. In contrast, the Pt/C electrode surface consistently maintains a large contact angle (Figure 4b), exhibiting distinct hydrophobic properties. To clarify the origin of the observed wettability, dynamic contact angle tests were extended to bare CF and Cu_3_P NWs@CF (Figure S28). Bare CF exhibits better wettability than the hydrophobic Pt/C@CF, which is constrained by the presence of hydrophobic polymer binders. This enhanced spreading on CF is further assisted by the reaction between surface copper oxides and the electrolyte, a process inhibited in Pt/C@CF due to the coating's shielding effect. Notably, Cu_3_P NWs@CF displays superhydrophilic behavior identical to that of PtCu/Cu_3_P NWs@CF. These findings indicate that the superhydrophilicity primarily originates from the self‐supporting nanowire array architecture, which markedly enhances surface roughness and capillary effects, rather than from the 3D porous CF substrate, Pt incorporation, or electrochemical activation. This pronounced hydrophilicity of PtCu/Cu_3_P NWs@CF facilitates efficient charge transfer at the electrode–electrolyte interface and promotes rapid electrolyte replenishment during operation at high current densities [10, 46].

For practical HER catalyst applications, operational stability at high currents is critical. To evaluate this, we conducted chronopotentiometry (CP) and amperometric *I*–*T* measurements at 1000 mA cm^−2^. Figure 4c displays that PtCu/Cu_3_P NWs@CF delivers relatively stable performance without obvious potential attenuation over 245 h of CP electrolysis, and the collected *I*–*T* data further verify its stable operation for more than 140 h under the same high‐current condition (Figure S29). Under identical conditions, Pt/C displays obvious degradation in a short time. In addition, Nyquist plots at different stages of the CP test reveal no obvious changes in the high‐frequency region, indicating that the solution resistance (R_s_) remains essentially stable during prolonged operation (Figure S30). Although the impedance arc becomes larger than that of the initial state, it does not continue to increase markedly with further electrolysis time, suggesting that the R_ct_ tends to stabilize rather than continuously deteriorate during prolonged operation, which further confirms the good electrochemical stability of PtCu/Cu_3_P NWs@CF under sustained high‐current conditions. Impressively, in acidic media, the stability of PtCu/Cu_3_P NWs@CF surpasses that of most advanced platinum‐based HER electrocatalysts reported to date (Figure 4d and Table S2), which endows it as a highly promising candidate for acidic water electrolysis.

The cycling stability of PtCu/Cu_3_P NWs@CF was also verified via 20 000 CV cycles (Figure S31). The polarization curve exhibits only a slight negative shift relative to the initial state over a wide current‐density range, confirming its excellent electrochemical robustness for high‐current acidic hydrogen production. To further evaluate the selectivity of hydrogen evolution, the Faraday efficiency (FE) of the PtCu/Cu_3_P NWs@CF catalyst was measured using a typical water drainage method in an H‐type electrolyzer. As shown in Figures S32 and S33, the volumes of H_2_ and O_2_ increase linearly with electrolysis time, indicating a steady and stable gas evolution process. Notably, the experimentally collected gas volumes exhibit a nearly ideal H_2_/O_2_ ratio of ∼2:1 (e.g., ∼4.7 mL H_2_ and ∼2.4 mL O_2_ at 1600 s), which is in agreement with the theoretical stoichiometry of water splitting. These results confirm that the FE for hydrogen evolution is close to 100%, demonstrating the high selectivity of the catalyst toward H_2_ production.

Meanwhile, we investigated the morphology and structure of PtCu/Cu_3_P NWs@CF after HER. The SEM images reveal abundant stacked pores on the surface of enoki‐like nanowires (Figure 4e and Figure S34), which remain firmly attached to the CF substrate without obvious peeling. This indicates a strong interfacial integration between PtCu/Cu_3_P NWs@CF and the CF substrate. In addition, it also implies that the active species underwent an inherent reconstruction process of dissolution‐redeposition during electrolysis, which leads to a porous enoki‐like morphology of PtCu/Cu_3_P NWs@CF nanowires. Such a hierarchical porous structure is highly favorable for rapid mass transfer under high‐current conditions. Furthermore, the TEM images demonstrate the reconstructed surface morphology (Figure 4f and Figure S35), showing that the loose surface of PtCu/Cu_3_P NWs@CF is composed of abundant nanoparticles. The HRTEM analysis identifies lattice stripes with a spacing of 0.190 and 0.251 nm, which correspond to the (200) and (111) planes of PtCu alloy and Cu_2_O, respectively (Figure 4g), indicating a reserved microstructure after the stability test. However, commercial Pt/C shows severe agglomeration only after 16 h of electrolysis (Figure 4h and Figure S36). Moreover, post‐stability XPS analysis shows negligible valence state change in the Cu 2p and Pt 4f spectra of PtCu/Cu_3_P NWs@CF (Figure 4i,j). Collectively, these findings suggest that PtCu/Cu_3_P NWs@CF undergo dynamic surface reconstruction during acidic HER (Figure 4k), which allows them to maintain an exceptional stability even at ampere‐level current density.

To provide direct experimental evidence for the proposed dynamic dissolution‐redeposition equilibrium, the concentration evolution of Cu and Pt ions in the electrolyte was monitored via offline inductively coupled plasma mass spectrometry (ICP‐MS) during electrolysis at 1000 mA cm^−2^ for PtCu/Cu_3_P NWs@CF. As summarized in Table S3 and illustrated in Figure S37, rather than the cumulative increase expected from the conventional leaching process, the concentrations of both Pt and Cu ions exhibited a pronounced downward trend and eventually reached a steady‐state plateau. For instance, the Pt concentration decreased from 0.1197 to 0.0486 mg L^−1^ during the tested period. This reduction in ion concentration during electrolysis unequivocally confirms that the dissolved metal species undergo electrochemical redeposition onto the cathode surface. The stabilization of these concentrations indicates that the rate of metal dissolution has reached a dynamic equilibrium with the rate of electrochemical redeposition. Such a dynamic dissolution‐redeposition equilibrium enables the maintenance of a stable, loose surface that is essential for the long‐term operational durability of PtCu/Cu_3_P NWs@CF at high current densities.

**FIGURE 5:**
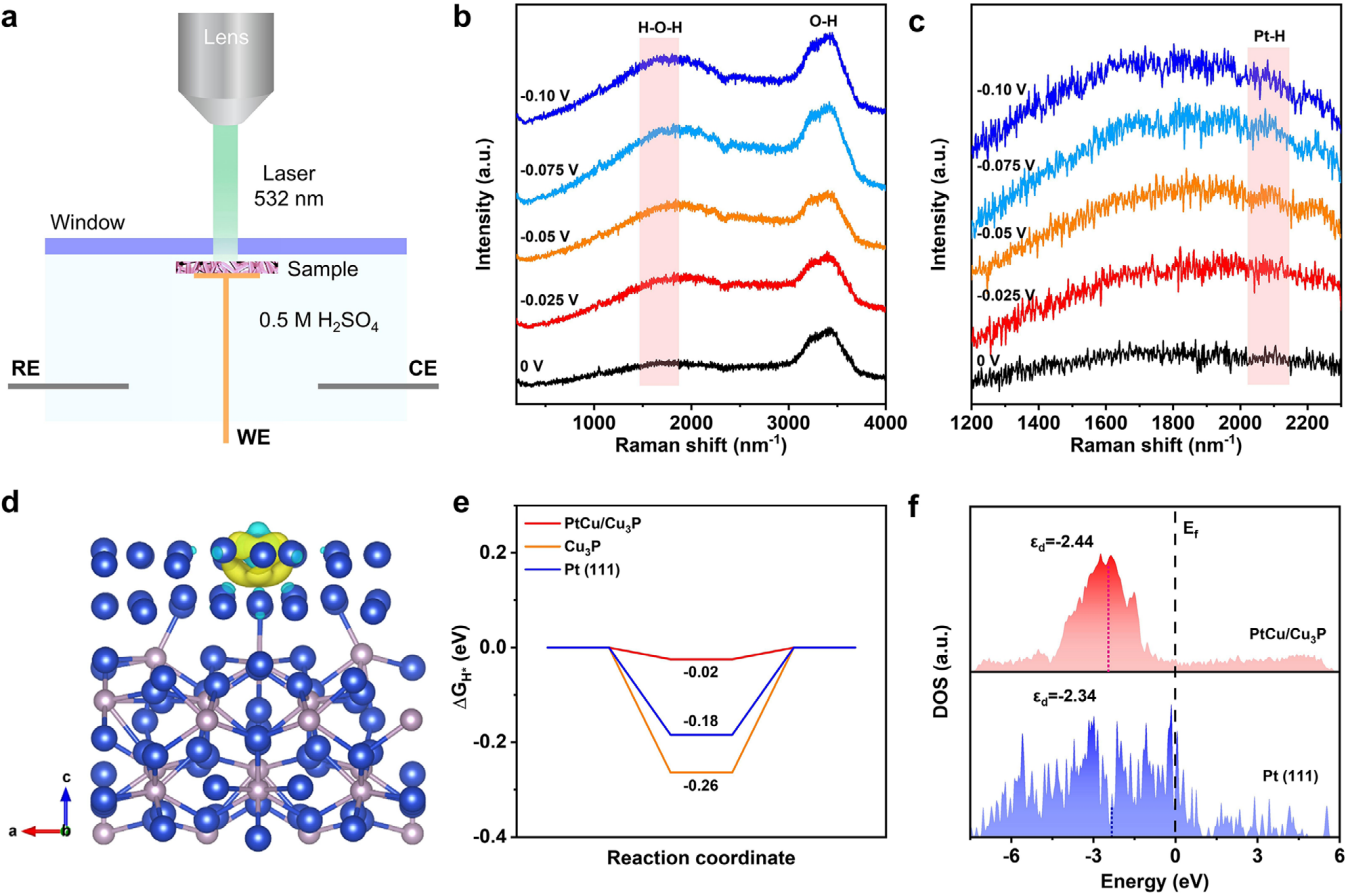
(a) Schematic of in situ Raman cell configuration (WE, working electrode; CE, counter electrode; RE, reference electrode). (b, c) In situ Raman spectra of PtCu/Cu_3_P NWs@CF at a series of applied potentials in 0.5 m H_2_SO_4_. (d) Side view of differential charge density for the PtCu/Cu_3_P heterostructure. Yellow and cyan areas denote electron depletion and accumulation, respectively. (e) H adsorption energy of PtCu/Cu_3_P, Cu_3_P, and Pt. (f) The DOS and analysis corresponding to the band center on PtCu/Cu_3_P and Pt (111), respectively.

In situ Raman spectroscopy was carried out to gain insight into the evolution of catalyst surface species and their chemical bonding during acidic HER (Figure 5a). The spectra reveal a broad feature spanning 3000–3600 cm^−1^ (Figure 5b), characteristic of the O─H stretching mode (ν_O─H_) of H_2_O molecule [47]. In Figure 5c, the peak appearing in the range of 2050–2100 cm^−1^ corresponds to the vibration mode of hydrogen atoms adsorbed on the Pt surface (ν_Pt‐H_) [48]. The peak intensity markedly increases with increasing potential, indicating that terminally adsorbed hydrogen serves as a key intermediate of HER [48, 49, 50]. Concurrently, the peak centered at around 1635 cm^−1^ belongs to the H─O─H bending vibration (δ_H─O─H_) of interfacial water molecules [50]. The peak intensity of δ_H─O─H_ enhances with more negative applied potentials, demonstrating strong hydrogen‐bonding interactions between adsorbed H atoms and neighboring interfacial water molecules [47]. These spectroscopic observations suggest that hydrogen atoms are adsorbed as the first layer on PtCu/Cu_3_P NWs@CF, while interfacial water molecules adsorb at the second layer. In this configuration, the interfacial water acts as a proton acceptor, forming hydrogen bonds with the adsorbed H atoms (H_3_O^+^). In contrast to Cu_3_P NWs@CF (Figure S38), the missing peaks of δ_H─O─H_ and the absorbed H imply a slow proton transfer kinetics during HER.

To further elucidate the catalytic mechanism, we investigated the influence of the heterostructure on the electronic structure and HER activity of the catalyst via density functional theory (DFT) calculations. Charge density difference analysis reveals (Figure 5d and Figure S39) a distinct electron aggregation at the Pt sites within the PtCu/Cu_3_P heterostructure, indicating that Pt primarily functions as an electron acceptor in this system. This electronic structure modulation is expected to influence the adsorption behavior of reaction intermediates, which can be evaluated by calculating the Gibbs free energy of hydrogen adsorption (ΔG_H*_) (Figure S40). Generally, a ΔG_H*_ closer to zero corresponds to more ideal HER activity [51, 52]. As shown in Figure 5e and Table S4, PtCu/Cu_3_P exhibits a ΔG_H*_ of −0.02 eV, outperforming Pt (111) (−0.18 eV) and Cu_3_P (−0.26 eV). This result indicates that PtCu/Cu_3_P exhibits superior hydrogen adsorption/desorption kinetics and higher intrinsic HER activity. Furthermore, the d‐band center (ɛ_d_) of a catalyst is a key descriptor determining its interaction strength with intermediates [11]. Density of states (DOS) calculations reveal that PtCu/Cu_3_P exhibits an ɛ_d_ of −2.44 eV, shifted to a lower energy level compared to Pt (111) (−2.34 eV). This downward shift lowers the anti‐bonding energy level relative to the Fermi level, weakening the interaction between the adsorbate and the surface and thereby reducing hydrogen‐binding strength on the surface of PtCu/Cu_3_P.

## 3 Conclusion

In summary, we successfully constructed a PtCu/Cu_3_P heterostructure on self‐supporting Cu_3_P NWs using a dynamic reconstruction method. This unique reconstruction process was initiated by electrochemically activating using a platinum counter electrode in an acidic electrolyte, yielding a HER cathode with high activity and outstanding stability. The resulting heterojunction optimizes H* adsorption energies and accelerates proton transfer kinetics, as confirmed by in situ Raman spectroscopy and DFT calculations, while its loose porosity in the restructured layer enables exceptional mass transport. As a result, the obtained electrode shows brilliant HER performance in acidic conditions, which can acquire 10 and 1000 mA cm^−2^ at ultralow overpotentials of 37.8 and 207.8 mV, respectively. More importantly, the HER performance is stable for over 240 h at 1000 mA cm^−2^, outperforming commercial Pt/C. The exceptional stability is likely attributed to a dynamic “dissolution‐redeposition” equilibrium mechanism on the surface of the restructured catalyst. Overall, this work provides fundamental insights into reconstruction chemistry in acidic environments and establishes a promising route for developing high‐performance cathodes for PEM electrolyzers.

## 4 Experimental Section

Detailed experimental procedures can be found in the Supporting Information.

## Author Contributions

K. N. H.: conceptualization, formal analysis, supervision, funding acquisition, project administration, resources, writing – review, and editing. C. Y.: visualization, formal analysis, data curation, writing – review, and editing. Y. Z.: methodology, data curation, investigation, validation, writing – review, and editing. K. W.: methodology, investigation, validation, data curation, formal analysis, writing – original draft, and conceptualization.

## Conflicts of Interest

The authors declare no conflicts of interest.

## Supporting information




**Supporting File**: advs76032‐sup‐0001‐SuppMat.docx.

## Data Availability

The data that support the findings of this study are available from the corresponding author upon reasonable request.
