# Supplementary material for: Dynamic Reconstruction‐Engineered Heterointerfaces for Acidic Hydrogen Evolution at Ampere‐Level Current Density
Source: Adv Sci (Weinh). 2026 Jun 12;13(50):e76032. doi: 10.1002/advs.76032 (PMC13336671; doi:10.1002/advs.76032)
Supplement: Supplementary file 1 [file ADVS-13-e76032-s001.docx]

**Supporting Information**

**Dynamic Reconstruction-Engineered Heterointerfaces for Acidic Hydrogen Evolution at Ampere-Level Current Density**

Table of Contents

1. Experimental Section
2. Supplementary Figures and Tables
3. Computational Section
4. References
1. **Experimental Section**

**Chemicals**

Ammonium persulfate ((NH_4_)_2_S_2_O_8_, ≥ 98%, AR, Sinopharm Chemical Reagent Co., Ltd.), sodium hydroxide (NaOH, 99.99%, metals basis, Aladdin Co.), sodium hypophosphite monohydrate (NaH_2_PO_2_, 99%, AR, Macklin Co.), hydrochloric acid (HCl, 37%, Aladdin Co.), sulfuric acid (H_2_SO_4_, GR, Sinopharm Chemical Reagent Co., Ltd.), acetone (AR, Sinopharm Chemical Reagent Co., Ltd.), ethanol (AR, Sinopharm Chemical Reagent Co., Ltd.), Nafion (5% w/w in water and 1-propanol, Dupont), commercial 20 wt% Pt/C (Shanghai Hesen Electric Co., Ltd.). All chemicals used in this experiment were used as received without further purification. Ultrapure water with the resistance of 18.25 MΩ cm^-1^ was used throughout all experiments.

**Preparation of Cu(OH)_2_ NWs@CF**

Copper foam (CF) was ultrasonically cleaned with acetone, ethanol, and ultrapure water before use. Three pieces of CF (2.0 × 2.5 cm^2^) were immersed in hydrochloric acid (10 mL, 0.1 M) for 1 h, followed by washing quickly with plenty of water. The obtained CF was then immersed in a mixed solution that containing 296.7 mg of (NH_4_)_2_S_2_O_8_ and 1.0 g of NaOH, and 10 ml water for 30 min at room temperature. After reaction, Cu(OH)_2_ NWs@CF was obtained after washing with water and ethanol several times and drying in a vacuum oven.

**Preparation of Cu_3_P NWs@CF**

Cu_3_P NWs@CF was prepared by low-temperature phosphating in a three-temperature zone tube furnace. Typically, 0.13 g of NaH_2_PO_2_∙H_2_O powder and one piece of Cu(OH)_2_ NWs@CF were placed on the upstream and downstream of the quartz tube at zone 1 and 3, respectively. The distance between NaH_2_PO_2_ and Cu(OH)_2_ NWs@CF is approximately 40 cm. Then, the zone 1 was heated to 290 ^o^C with a ramp rate of 10 ^o^C min^−1^ and maintained at 290 ^o^C for 1 h under N_2_ atmosphere, but zone 2 and zone 3 were procedurally kept at room temperature. After cooling to room temperature, the Cu_3_P NWs@CF was harvested. Additionally, a series of control experiments were performed. Specifically, the corresponding phosphorization products with using 0.07 g and 0.19 g of NaH_2_PO_2_ were prepared (labeled as Cu_3_P NWs@CF-0.07g and Cu_3_P NWs@CF-0.19g). The Cu(OH)_2_ NWs@CF was treated with the phosphorization distance of 20 cm and 2 cm and then labeled as Cu_3_P NWs@CF-20cm and Cu_3_P NWs@CF-2cm.

**Preparation of PtCu/Cu_3_P NWs@CF**

PtCu/Cu_3_P NWs@CF was obtained by electrochemical activation of Cu_3_P NWs@CF in 0.5 M H_2_SO_4_ under a standard three-electrode system with a 0.5 × 0.5 cm^2^ Cu_3_P NWs@CF as the working electrode, a 1.5 × 1.5 cm^2^ platinum foil and Ag/AgCl electrode as the counter and reference electrodes, respectively. The electrochemical activation was conducted with the chronopotentiometry at a constant current density of 2000 mA cm^−2^ for 50 h, resulting in the formation of PtCu/Cu_3_P NWs@CF.

**Characterizations**

Scanning electron microscope (SEM) images were taken to investigate the morphology of the as-synthesized samples using a field emission scanning electron microscope (FESEM, JEOL JSM-7500FA). Transmission electron microscopy (TEM) images were obtained by a JEOL JEM-2100 electron microscope working at 200 kV. Powder X-ray diffraction (PXRD) patterns were taken on a Rigaku Smartlab 9000W diffractometer with Cu Kα radiation (λ = 0.15418 nm) operating at 40 KV and 200 mA. X-ray photoelectron spectra (XPS) patterns were collected by a Thermo ESCALAB 250 spectrometer with a monochromatic Al Kα as the excitation source. Raman spectra were collected on a Micro Raman System (Horiba LABHRev-UV) at an excitation wavelength of 532 nm. The element content of the catalyst was evaluated by an inductively coupled plasma-atomic emission spectrometry (ICP-AES, IRIS(HR)).

**In-situ Raman measurement**

A Micro Raman System (Horiba LABHRev-UV) with an excitation wavelength of 532 nm and a cell with a quartz window (Wuhan Gaoss Union Technology Co., LTD) were employed for the in-situ Raman measurements. The obtained catalyst, a piece of Pt foil, and Ag/AgCl were used as the working electrode, counter electrode, and reference electrode, respectively. The laser beams were perpendicularly focused on the catalyst and the backscattered light was collected through the quartz window. The chronoamperometry method was used to apply different voltages to the electrode at 25 mV intervals over a voltage range of 0 to -0.1 V vs. RHE.

**Electrocatalytic measurement**

All the electrochemical measurements were carried out on a Bio-Logic EC-LAB (VMP-300) equipment at room temperature. The acidic HER performance in 0.5 M H_2_SO_4_ were performed under a standard three-electrode system, in which the fabricated catalysts were directly employed as the working electrode (0.5 × 0.5 cm^2^) without any binder, a carbon rod was used as the counter electrode, and a Ag/AgCl electrode was used as the reference electrode. The potentials were converted to reversible hydrogen electrode (RHE) according to the relation: E_RHE_ = E_Ag/AgCl_ + 0.059pH + 0.197. The linear sweep voltammetry (LSV) curves were conducted at a scan rate of 5 mV s^−1^. The Tafel plots could be obtained by plotting overpotential (*η*) vs. log current density (log *j*) using LSV curves. Then fitting the linear portion of the Tafel plots to get the Tafel slope (b) according to the Tafel equation: *η* = blog (*j*) + a.

The electrochemical active surface area (ECSA) of the catalysts was estimated by measuring the electrochemical double-layer capacitance (C_dl_) using cyclic voltammetry (CV) curves under a potential window of 0.047 ~ 0.147 V vs. RHE at scanning rates of 20, 40, 60, 80, and 100 mV s^−1^ in the non-Faradaic region. The C_dl_ were equivalent to the half linear slope, which was obtained by plotting the difference of current density between the anodic and cathodic at 0.097 V versus RHE against the scan rate. ECSA was calculated as follows: ECSA= C_dl_ (*catalyst*)/(C_dl_ (*CF*)∙*per ECSA cm^−2^*). Here, the S_geo_ represents the geometric surface area of the working electrode, and C_s_ is specific electrochemical double-layer capacitance and its value is 0.040 mF cm^−2^.

Electrochemical impedance spectroscopy (EIS) measurements were carried out at the overpotential of 81 mV with a frequency range from 10^4^ to 0.1 Hz with an amplitude of 5 mV. All the measured polarization curves in this work were iR-corrected by the equation: E_cor_ = E – iR_s_, where E_cor_ was the iR-corrected potential, E was the measured potential (vs. RHE), i was the current and R_s_ was the internal resistance (the resistance of the electrolyte/contact between the reference and working electrode) derived from EIS plot. TOF was calculated according to the equation: TOF = j*(η)*A/nFN, here, j was the current density at a certain overpotential of *η* with iR-correction, A was the geometric area of the electrode (0.25 cm^−2^), n was the number of electron transfer per molecule in the reaction (2 for HER), F was the Faraday constant (96485 C·mol^−1^), and N was the mole number of the active site. Assuming that all Pt metal atoms on the electrode serve as active sites, which were determined by ICP-OES. The long durability tests were performed at constant current density of 1000 mA cm^−2^. Accelerated durability tests (ADT) were conducted via continuous CV scanning for 20000 cycles at 100 mV s^−1^ within a potential window of -0.5 V to -0.2 V vs. Ag/AgCl. The Faraday efficiency (FE) was analyzed by the water drainage method using an H-type electrolyzer.

For the preparation of Pt/C electrode, 5 mg of the catalyst powder was dispersed in a 500 µL mixed solution of ethanol, water and Nafion solution (5 wt%) with a volume ratio of 2:2:1 to form a homogeneous ink. Then, the obtained ink was pipetted onto a piece of 0.5 × 1 cm^2^ copper foam with the coating area of 0.25 cm^2^ (the catalyst loading: 0.5 mg cm^-2^) and dried in air to form the working electrode.

2. **Supplementary Figures and Tables**

**Figure S1.** (a-c) SEM images at different magnifications and (d) XRD pattern of Cu foam (denoted as CF).

**Figure S2.** (a-c) SEM images at different magnifications and (d) XRD pattern of Cu(OH)_2_ NWs@CF.

**Figure S3.** (a, b) TEM images, and (c) HRTEM image of Cu(OH)_2_ NWs@CF. Inset in (c) is the HRTEM image at higher magnification from the dashed red area.

**Figure S4.** (a) Schematic representation of the low-temperature phosphating step. (b) Digital photo of the maximum temperature in the phosphating process in different temperature zones of the tube furnace.

**Figure S5.** (a-c) SEM images at different magnifications and (d) XRD pattern of Cu_3_P NWs@CF.

**Figure S6.** (a, b, c) TEM images, and (d) HRTEM image of Cu_3_P NWs@CF. Inset in (d) is the HRTEM image at higher magnification from the dashed red area.

**Figure S7.** (a) The full XPS survey spectrum, (b) High-resolution Cu 2p XPS spectra, and (c) P 2p XPS spectra of Cu_3_P NWs@CF.

**Figure S8.** SEM images of (a-c) Cu_3_P NWs@CF-0.07g, (d-f) Cu_3_P NWs@CF-0.19g, (g-i) Cu_3_P NWs@CF-20cm, and (j-l) Cu_3_P NWs@CF-2cm.

**Figure S9.** XRD pattern of various Cu_3_P NWs@CF obtained at different phosphating conditions.

**Figure S10.** (a) HER polarization curves, (b) Corresponding overpotentials at 10 mA cm^−2^, and (c) Corresponding Nyquist plots of various Cu_3_P NWs@CF obtained at different phosphating conditions.

**Figure S11.** Schematic representation of the electrochemical activation step.

**Figure S12.** Digital photo of pH test for 0.5 M H_2_SO_4_.

**Figure S13.** SEM images of PtCu/Cu_3_P NWs@CF at different magnifications.

**Figure S14.** Chronopotentiometry response of Cu_3_P NWs@CF at a constant current density of 2000 mA cm^−2^ with a graphite rod as the counter electrode.

**Figure S15.** SEM images of Cu_3_P NWs@CF-Gr.

**Figure S16.** (a, b) XRD patterns of PtCu/Cu_3_P NWs@CF and PtCu/Cu_3_P NWs@CF powder exfoliated from the bulk sample, respectively.

**Figure S17.** TEM image of PtCu/Cu_3_P NWs@CF.

**Figure S18.** (a) The full XPS survey spectrum, (b) High-resolution P 2p XPS spectra, and (c) O 1s XPS spectra of PtCu/Cu_3_P NWs@CF.

**Figure S19.** (a) XRD patterns and (b) corresponding magnified patterns of the Cu_3_P NWs@CF electrodes with different activation times (the samples are denoted as Cu_3_P NWs@CF-X, where X refers to the activation time).

**Figure S20.** SEM images of (a, b) Cu_3_P NWs@CF-5h, (c, d) Cu_3_P NWs@CF-20h, and Cu_3_P NWs@CF-35h.

**Figure S21.** (a, c, e) TEM images and (b, d, f) HAADF-STEM with corresponding EDS elemental mapping images of Cu_3_P NWs@CF-5h, Cu_3_P NWs@CF-20h, and Cu_3_P NWs@CF-35h, respectively.

**Figure S22.** (a) HER polarization curves, (b) Corresponding overpotentials at 10 mA cm^−2^, and (c) Corresponding Nyquist plots of the Cu_3_P NWs@CF electrodes with different activation times.

**Figure S23.** Mass activities of PtCu/Cu_3_P NWs@CF and Pt/C.

**Figure S24.** CV Curves of (a) PtCu/Cu_3_P NWs@CF, (b) Cu_3_P NWs@CF, (c) CF, and (d) Pt/C at different scan rates.

**Figure S25.** Double-layer capacitance (C_dl_) plots of PtCu/Cu_3_P NWs@CF, Cu_3_P NWs@CF, CF and Pt/C.

**Figure S26.** (a) The calculated electrochemically active surface area (ECSA), and (b) ECSA normalized LSV curves of different samples.

**Figure S27.** The TOF at the overpotential of η = 0.2 V.

**Figure S28.** Dynamic contact angle measurement for (a) Cu foam and (b) Cu_3_P NWs@CF in 0.5 M H_2_SO_4_.

**Figure S29.** The amperometric i-t curve of PtCu/Cu_3_P NWs@CF at around 1000 mA cm^−2^.

**Figure S30.** Nyquist plots of PtCu/Cu_3_P NWs@CF collected after different operation times at 1000 mA cm^−2^.

**Figure S31.** (a) The polarization curves of PtCu/Cu_3_P NWs@CF before and after 20000 CV cycles, and (b) the overpotential increasements after 20000 CV cycles at different current densities.

**Figure S32.** (a) The corresponding levels of H_2_ and (b) O_2_ generated at different electrolysis times for PtCu/Cu_3_P NWs@CF||Pt foil at the current density of 100 mA cm^−2^.

**Figure S33.** The amount of H_2_ and O_2_ collected by the water drainage method as a function of time for PtCu/Cu_3_P NWs@CF||Pt foil.

**Figure S34.** SEM images of (a, b) the nanowire region, and (c, d) the region around nanowires in PtCu/Cu_3_P NWs@CF after stability test.

**Figure S35.** TEM images of PtCu/Cu_3_P NWs@CF after stability test.

**Figure S36.** (a-c) TEM images of commercial Pt/C before the stability test, and (d, e) TEM images after the stability test.

**Figure S37.** Time-dependent evolution of the Pt and Cu ions concentration in the electrolyte during HER electrolysis at 1000 mA cm^−2^ in 0.5 M H_2_SO_4_.

**Figure S38.** In-situ Raman spectra of Cu_3_P NWs@CF in 0.5 M H_2_SO_4_ at a series of applied potentials.

**Figure S39.** Top view of differential charge density difference in PtCu/Cu_3_P. Yellow and cyan areas represent electron depletion and accumulation.

**Figure S40.** Different adsorption position of H on (a) Pt (111), (b) Cu_3_P (112), and (3) PtCu/Cu_3_P. The gray, blue, and purple spheres represent Pt, Cu, and P atoms, respectively.

**Table S1.** Normalized atomic percentages of Cu and Pt and corresponding Pt/Cu atomic ratios derived from SEM-EDS analysis for the Cu_3_P NWs@CF electrodes with different activation times.

| **Sample** | **Cu (at.%)** | **Pt (at.%)** | **Pt/Cu (atomic ratio, %)** |
| --- | --- | --- | --- |
| Cu_3_P NWs@CF-5h | 99.39 | 0.61 | 0.615 |
| Cu_3_P NWs@CF-20h | 99.09 | 0.91 | 0.919 |
| Cu_3_P NWs@CF-35h | 98.79 | 1.21 | 1.225 |

**Table S2.** Comparison of recent reported high-performance Pt-based electrocatalysts for HER in 0.5 M H_2_SO_4_.

| **No.** | **Electrocatalysts** | **η_10_**  **(mV)** | **η_50_**  **(mV)** | **η_100_**  **(mV)** | **η_200_**  **(mV)** | **η_500_**  **(mV)** | **Stability** | **Reference** |
| --- | --- | --- | --- | --- | --- | --- | --- | --- |
| 1 | PtCu/Cu_3_P NWs@CF | 37.8 | 79.0 | 101.8 | 128.8 | 174.0 | CP (1000 mA cm^-2^): 245 h | **This Work** |
| 2 | Pt/TiO_2_-O_V_ | 18 | - | - | - | - | CA (~10 mA cm^-2^): 20 h | Angew. Chem. Int. Ed. **2023**, 62, e202300406 |
| 3 | PtSe_2_ nanosheets | ~45 | ~117 | - | - | - | CP (200, 500 mA cm^-2^): ~150 h | ACS Energy Lett. **2022**, 7, 3675 |
| 4 | PtCu/WO_3_@CF | 41 | ~110 | ~140 | ~165 | ~190 (400 mA cm^-2^) | CP (20 mA cm^-2^): 20 h | Adv. Funct. Mater. **2022**, 32, 2112207 |
| 5 | Pt/TiB_x_O_y_ | ~50 | 98 (40 mA cm^-2^) | - | - | - | CA (~10 mA cm^-2^): 10 h | ACS Catal. **2022**, 12, 5970 |
| 6 | Pt@Cu-0.3 | 67 | ~172 | - | - | - | - | Adv. Funct. Mater. **2021**, 31, 2105579 |
| 7 | Pt_1_/NMHCS | 40 | ~116 | - | - | - | CP (10 mA cm^-2^): 10 h | Adv. Mater. **2021**, 33, 2008599 |
| 8 | Pt-SAs/WS_2_ | 32 | ~108 (60 mA cm^-2^) | ~215 (120 mA cm^-2^) | - | - | - | Nat. Commun. **2021**, 12, 3021 |
| 9 | Pt/RuCeO_x_-PA | 41 | - | - | - | - | CP (10, 20, 30, 40, 50 mA cm^-2^): 8 h | Angew. Chem. Int. Ed. **2020**, 59, 20423 |
| 10 | a-Pt_3_V | 20 | ~80 | ~101 | ~145 | ~215 | CP (500 mA cm^-2^): 100 h | Adv. Energy Mater. **2023**, 13, 2300127 |
| 11 | Pt-CoP | 79 | 113 | ~133 | 200 (300 mA cm^-2^) | - | CP (100 mA cm^-2^): 30 h | Adv. Sci. **2025**, 12, 2504462 |
| 12 | Pt@VNC | 5 | - | 33 | - | - | CA (10 mA cm^-2^): 40 h  CA (200 mA cm^-2^): 85% after 100 h | Adv. Energy Mater. **2023**, 13, 2204213 |
| 13 | Pt-Ru(fcc)/C | 4 | - | ~140 (150 mA cm^-2^) | - | - | CA (50 mA cm^-2^): 20 h | Adv. Mater. **2025**, 37, 2503221 |
| 14 | Pt-TiO_2_/CC | 38 | - | 88 | - | 183 (400 mA cm^-2^) | CA (35 mV): 65 h | Appl. Surf. Sci. **2025**, 687, 162257 |
| 15 | Pt_1_-PPy | 45.2 | - | 150 | - | - | - | Adv. Funct. Mater. **2024**, 34, 2404707 |
| 16 | 2D PhenPtCl_2_ | 41 | - | - | - | - | CA (10 mA cm^-2^): 10 h | Nat. Commun. **2024**, 15, 385 |
| 17 | Pt-MoC/NCT | 74 | - | 286 | - | - | CA (10 mA cm^-2^): 100 h | Chin. Chem. Lett. **2025**, 36, 109713 |
| 18 | Pt SAs/Mo-PtNi/C | 38 | - | - | - | - | CA (60 mA cm^-2^): 12 h | Fuel **2025**, 381, 133356 |
| 19 | Pt/PMx | ~125 | 350 | - | - | - | CA (10 mA cm^-2^): 40 h | J. Mater. Chem. A **2025**, 13, 36550 |
| 20 | PtRu-Co_3_O_4_ | 99 | - | 116 | - | - | CP (50 mA cm^-2^): 68 h | J. Am. Chem. Soc. **2024**, 146, 28728 |
| 21 | Pt_x_/TiO_2_ NTs@3D-Ti | 53 | - | 200 | - | - | CP (20 mA cm^-2^): 20 h | Mater. Today Energy **2022**, 27, 101042 |
| 22 | Ag@Pt icosahedral NCs | 35 | - | 70 | - | - | CP (1000 mA cm^-2^): 100 h | Compos. Part B **2023**, 254, 110600 |
| 23 | Pt/HE-LDH | 42 | ~172 (70 mA cm^-2^) | - | - | - | CP (10 mA cm^-2^): 22 h | J. Colloid Interface Sci. **2025**, 684, 566 |
| 24 | Pt_1_/(CoNi@Gr) | 12 | - | ~103 | - | - | CP (10 mA cm^-2^): 90 h | Joule **2025**, 9, 101968 |
| 25 | m-Pt@MoS_2_ | 47 | ~90 | ~122 (75 mA cm^-2^) | - | - | CA (50 mV): 12 h | Small **2024**, 20, 2309427 |
| 26 | Pt SAs/MoO_2_ NRs | 9.3 | ~43 | - | ~162 | - | CP (1000 mA cm^-2^): 200 h | Chem. Eng. J **2022**, 427, 131309 |

**Table S3.** Corrected off-line inductively coupled plasma mass spectrometry (ICP-MS) concentrations of dissolved Cu and Pt species in the electrolyte during HER electrolysis.

| **Electrolysis time (h)** | **Pt concentration (mg L^−1^)** | **Cu concentration (mg L^−1^)** |
| --- | --- | --- |
| 2 | 0.1197 | 1.6819 |
| 2.5 | 0.0727 | 1.6876 |
| 3.0 | 0.0663 | 1.2659 |
| 3.5 | 0.0491 | 1.3282 |
| 4.0 | 0.0486 | 1.0403 |

Note: PtCu/Cu_3_P NWs@CF was first electrolyzed in 30 mL of 0.5 M H_2_SO_4_ at 1000 mA cm^−2^ for 2 h before the first sampling. At each sampling point, 1 mL electrolyte was withdrawn and immediately replenished with 1 mL fresh blank electrolyte to maintain a constant total volume. Electrolysis was then continued for another 0.5 h before the next sampling step. A total of five sampling cycles were conducted.

**Table S4**. DFT-calculated energy parameters for hydrogen adsorption on PtCu/Cu_3_P, Cu_3_P, and Pt (111) surfaces at 298.15 K.

| **Sample** | **E_(System)_ (eV)** | **E_(System + H*)_ (eV)** | **E_ZPE_ (eV)** | **ΔS (eV K^-1^)** | **T (K)** | **∆G_H*_ (eV)** |
| --- | --- | --- | --- | --- | --- | --- |
| PtCu/Cu_3_P | -392.74447 | -396.39197 | 0.04518 | 0.00013 | 298.15 | -0.02 |
| Cu_3_P | -189.38552 | -193.27261 | 0.06697 | 0.00021 | 298.15 | -0.26 |
| Pt (111) | -184.70763 | -188.43565 | 0.055684 | 0.00044 | 298.15 | -0.18 |

Note: The Gibbs free energy of hydrogen adsorption (ΔG_H*_) was calculated using the standard formula: ΔG_H*_ = (E_(System + H*)_ - E_(System)_ - 1/2E_H2_) + ΔE_ZPE_ - TΔS, where E_(System + H*)_ and E_(System)_ are the total energies of the surface with and without adsorbed H, respectively; E_H2_ is the energy of gas-phase H_2_ molecule; ΔE_ZPE_ is the zero-point energy correction; T is the temperature; and ΔS is the entropy difference between adsorbed H and gas-phase H_2_.

3. **Computational Section**

All the calculations were carried out on the basis of the spin-polarized density functional theory DFT^[2]^ methods within general gradient approximation parametrized by Perdew, Burke, and Ernzerhof.^[3]^ A Monkhorst-Pack grid of 2 × 2 × 1 and 3 × 3 × 1 size was used to sample the Brillouin zone in calculations for NiO(200) and Pt(111), respectively.^[4]^ Structures were relaxed until the total forces were less than 0.02 eV/Å and the convergence criterion for total energies was set to 10^-5^ eV. Meanwhile, the DFT-D3 correction was used to describe the van der Waals interactions in the systems.^[5,6]^ A plane-wave energy cut-off of 450 eV was set, and the vacuum layer was 15 Å to avoid any artificial effect. Besides, the U schemes should be applied for Ni atoms, which were set as 3.0 eV.^[7]^ The HER catalytic activity of materials can be evaluated by ΔG_H*_, which is defined as

ΔG_H*_ = ΔE_H*_ + ΔE_ZPE_ − TΔS_H*_,

where ΔE_H*_ is the hydrogen adsorption energy defined as

ΔE_H*_ = E_(System + H*)_ − E_(System)_ −1/2 E_H2_,

in which E_(System + H)_ and E_(System)_ are the energies of all research systems with and without H adsorption, respectively. ΔE_ZPE_ is the amount of change of zero-point energy in the total system, and T is 298.15 K. ΔS_H_ is the entropy change of the H atom after it transits from gaseous to adsorbed state. Herein, ΔE_ZPE_ − TΔS_H_ is approximated to 0.24.^[8]^

4. **References**

[1]	X. Li, C. Liu, Z. Fang, L. Xu, C. Lu, W. Hou, *Small* **2022**, *18*, 2104354

[2]	S. Grimme, *J. Comput. Chem. Mater.* **2006**, *27*, 1787.

[3]	J. P. Perdew, K. Burke, M. Ernzerhof, *Phys. Rev. Lett.* **1996**, *77*, 3865.

[4]	H. J. Monkhorst, J. D. Pack, *Phys. Rev. B* **1976**, *13*, 5188.

[5]	S. Grimme, J. Antony, S. Ehrlich, H. Krieg, *J. Chem. Phys.* **2010**, *132*, 1

[6] S. Grimme, S. Ehrlich, L. Goerigk, *J. Comput. Chem.* **2011**, *32*, 1456.

[7]	F. H. Aragón, P. E. N. de Souza, J. A. H. Coaquira, P. Hidalgo, D. Gouvêa, *Physica B Condens. Matter.* **2012**, *407*, 2601.

[8]	J. K. Nørskov, T. Bligaard, A. Logadottir, J. R. Kitchin, J. G. Chen, S. Pandelov, U. Stimming, *J. Electrochem. Soc.* **2005**, *152*, J23.
